# ASSOCIATIONS BETWEEN SHARED TIME AND MENTAL HEALTH IN MARRIED COUPLES

**DOI:** 10.1093/geroni/igad104.1415

**Published:** 2023-12-21

**Authors:** Selin Karakose, Thomas Ledermann, Angelina Sutin, Antonio Terracciano

**Affiliations:** Florida State University College of Medicine, Tallahassee, Florida, United States; Florida State University, Tallahassee, Florida, United States; Florida State University College of Medicine, Tallahassee, Florida, United States; Florida State University, Tallahassee, Florida, United States

## Abstract

Spending time with the partner has been found to be beneficial in many ways for both the relationship and the individuals. However, little is known about the associations between time spend together and the partner’s mental health. The present study examined the dyadic associations between time spent with spouse and mental health, particularly depressive symptoms, anxiety, and stress. Using data from 145 married couples from Turkey (M wives = 39.84 years, M husbands = 42.97), we investigated whether time spent with the spouse is associated with the mental health of both the individual and their spouse. Actor-Partner Interdependence Model (APIM) analyses indicated that both husbands’ and wives’ perception of time spent together was associated with lower stress. Additionally, for wives, time spent together was negatively associated with their own depression. These findings revealed that spending time together can help to cope with stress and have a positive effect on wives with elevated depressive scores.

